# Monthly Summary

**Published:** 1881-08

**Authors:** 


### MONTHLY SUMMARY.

*Sensitive Dentine.*—One of the best means of overcoming this is by the application of the rubber dam. As a rule the *lighter* the color of the decay the *greater* will be the sensitiveness. If several cavities in the teeth are to be prepared for filling, and one is found more sensitive than another, begin work on the *least* sensitive after having applied the rubber dam. If *absolute* alcohol be applied to the sensitive cavities on a small bit of spunk or cotton, and left *in situ* until entirely evaporated, and the preparation of the insensitive cavity be accomplished, and even until entire filling with gold or other filling material, considerable absence of pain in the cavities thus treated will be found to have ensued. The cause of this is in the complete dryness that is induced, as well by application of the dam as by the application of the absolute alcohol; for as this (the alcohol) has a very great affinity for water, it robs the dentine of some of its natural moisture, and leaves it almost as dry as dead bone. If, on attempting to excavate, sensitiveness is still present, a little chloroform applied on a bit of spunk to the cavity will generally obtain an almost total immunity of pain. It may be, however, that the chloroform will affect only a certain depth of the sensitive dentine. so that several applications of the chloroform may have to be made before the cavity is of proper shape for the introduction of the filling. We know of no treatment so efficacious as this. Absolute dryness seems to be the best treatment, and this can only be obtained by means of the rubber dam, and the great affinity which alcohol has for water, and thus drying out the cavity to be excavated and filled.—*Dental Office and Laboratory.*

---

*To Prevent the Clouding of Mirrors by Moisture.*—The trouble may be simply effectually obviated, by passing lightly over the surface of the glass or speculum a cloth or sponge slightly moistened with glycerine. Glycerine has considerable avidity for moisture, and in consequence the vehicles of water vapor contained in the expired breath or in the moist atmosphere of the bath-room, in touching this surface are instantly dissolved, so that no cloud is formed. If the reader will try this simple receipt, he will find himself relieved of the annoyance alluded to, and will be able to shave in peace. We can particularly recommend this simple and effective remedy against the tarnishing of mirror surfaces by moisture to the medical profession, as vastly more practical and better than the practice now in vogue of dipping them in tepid water or heating slightly over a lamp before using their instruments, as by the simple use of glycerine in the manner we have described, there will be no fear of giving the patient an unpleasant or painful sensation by accidental overheating of the mirror when applied to sensitive or inflamed parts.—*Manufacturer and Builder.*

---

*Dangers of Dentistry.*—Usually dental surgeons take great care to keep their implements clean. Sometimes, however, the patient is disgusted with the sight of more or less ancient blood stains on forceps and other implements which are to go into his mouth.

A correspondent in Maine submits a local newspaper report of an accident to a Bangor dentist, which suggests the query whether there may not be danger of blood poisoning to the hazard of the patient's life when the surgeon is not careful with respect to the cleanliness of his implements. In the case reported the accidental pricking of a finger with a sharp instrument used by the dentist while filling a tooth, resulted in a serious case of pyæmia. In this instance the dentist was the sufferer. Suppose the poisoned tool had pricked the gum of the patient? Whether the poison came from the diseased tooth then being operated on, or was due to some previous operation, does not appear, and would not much matter to a patient who would be poisoned in that way. In either case the injury might be fatal. From a moral point of view, however, it would make a great difference whether the patient furnished the poison or the dentist. It goes without saying that untidiness in the dentist's chair is dangerous as well as disgusting, and should not be tolerated.—*Scientific American.*

*Analyzing Metals by Electrolysis.*—Perhaps the most important information presented to the Academy, during its earlier sessions at least, was Professor Wolcott Gibbs's new method of analyzing metals by electrolysis. His plan is to place the metal in solution in a beaker, add pure mercury, and connect the mercury with an electric battery. By the electric action the metal was thrown down upon the mercury and the beaker beforehand, and then after the process to determine the metal by again weighing the vessel and the mercury. This method, he said, was applicable to mercury, tin, cobalt, and other metals. It did not apply in arsenic and antimony. He did not despair of separating potassium and sodium by the process, although his experiments with these metals had not been completely successful.

Professor Hunt said this process came with the beauty and force of a revelation; its simplicity recommended it. Every chemist would await further developments with great interest. He also asked what battery power was used. Professor Gibbs said the power of the battery was immaterial, except in point of time. The stronger the power the shorter the time required for the process. With a power equal to a Bunsen battery of 40 or 50 cells he had precipitated 15 grammes of zinc from a solution in from 20 to 25 minutes. A battery power of two or three cells would probably precipitate 3 or 4 grammes of zinc in an hour.—*National Academy of Sciences, N. Y.*

---

*Dangers of "Bonwill Method" of Anæsthesia.*—This method, as is well known, is by means of forced respirations. Dr. Henry Gibbons, of San Francisco, says of it, in the *Pacific Med. Journal:*

"We suppose that most persons, most boys, at any rate, have experienced the sensation produced by blowing at a fire, substituting their lungs for a bellows. The proposed method is nothing more than an exaggeration of this. So far from regarding it as likely to prove useful, we look upon it as decidedly dangerous. It puts the brain in an abnormal condition, by interfering with the circulation, and is almost equivalent to partial strangulation. We are surprised that any considerate physician should recommend such a violation of physiological laws."

